# Knowledge, Attitudes, Behavioural Practises, and Psychological Impact Relating to COVID-19 Among People Living With Spinal Cord Injury During In-Patient Rehabilitation in Bangladesh

**DOI:** 10.3389/fneur.2021.739354

**Published:** 2022-02-07

**Authors:** Mohammad Anwar Hossain, K. M. Amran Hossain, Mohamed Sakel, Md. Feroz Kabir, Karen Saunders, Rafey Faruqui, Mohammad Sohrab Hossain, Zakir Uddin, Manzur Kader, Lori Maria Walton, Md. Obaidul Haque, Rubayet Shafin, Sonjit Kumar Chakrovorty, Iqbal Kabir Jahid

**Affiliations:** ^1^Department of Physiotherapy, Centre for the Rehabilitation of the Paralysed (CRP), Dhaka, Bangladesh; ^2^Department of Microbiology, Jashore University of Science and Technology (JUST), Jashore, Bangladesh; ^3^Department of Physiotherapy, Bangladesh Health Professions Institute (BHPI), Dhaka, Bangladesh; ^4^East Kent Hospitals University NHS Foundation Trust, Canterbury, United Kingdom; ^5^Department of Physiotherapy and Rehabilitation, Jashore University of Science and Technology (JUST), Jashore, Bangladesh; ^6^School of Social Policy, Sociology and Social Research, University of Kent, Canterbury, United Kingdom; ^7^Department of Neuropsychiatry, Kent & Medway NHS Social Care Partnership Trust, Maidstone, United Kingdom; ^8^Centre for Health Services Studies, University of Kent, Canterbury, United Kingdom; ^9^School of Rehabilitation Science, McMaster University, Hamilton, ON, Canada; ^10^Institute of Environmental Medicine, Karolinska Institute, Stockholm, Sweden; ^11^Department of Physical Therapy, School of Health Sciences, University of Scranton, Scranton, PA, United States

**Keywords:** spinal cord injury, knowledge attitudes behavioural practices, psychological impact, depression, anxiety, stress, COVID-19, Bangladesh

## Abstract

**Aim:**

The aim of this research is to focus on gaining an insight into the knowledge, attitudes, behavioural practises (KAP), and psychological impact relating to COVID-19 among the people living with spinal cord injury receiving in-patient rehabilitation.

**Methods:**

A prospective, cross-sectional survey of people with SCI (*N* = 207), who were in active in-patient rehabilitation from two tertiary SCI Rehabilitation Centres in Bangladesh. Data were collected via face-to-face interviews, after voluntary consent, using a pretested, language validated questionnaire on Knowledge, Attitude and Behavioural practises (KAP) and the Depression, Anxiety, Stress Scale (DASS-21). Ethical approval and trial registration were obtained prospectively.

**Results:**

A total of 207 people with SCI responded, among which 87% were men and 13% were women, with a mean age of 34.18 ± 12.9 years. Within the sample group, people living with tetraplegia comprised 33.8%, and people living with paraplegia comprised 66.2%. Overall, 63.8% of the participants were diagnosed with an SCI categorised as ASIA-A. Overall, the “knowledge score” was 8.59 ± 2.3 out of 12, “depression” was 11.18 ± 8, “anxiety” was 7.72 ± 5.1, and “stress” was 9.32 ± 6.7 from a total of 21 scores each category. The strong correlation was between knowledge, DASS scores, and age (*p* < 0.05). In addition, there was a strong correlation between knowledge, gender (*p* < 0.05) and education (*p* < 0.01). Binary logistic regression found a stronger association of knowledge and DASS scores with gender, young age, illiteracy (*p* < 0.01), and rural residence (*p* < 0.05). A positive relationship was found between depression and anxiety scores (*p* < 0.01) and a moderate positive relationship was found between depression and stress scores (*p* < 0.01). A positive attitude was reported by the majority of participants (*p* < 0.05). In terms of behavioural practises, participants reported both self and caregiver had followed health advice with regard to consulting health professionals (65.7%), implementing isolation (63.8%), taking droplet precaution care (87.4%), and hygiene care (90.3%).

**Conclusion:**

Participants in this study reported high levels of knowledge, adoption of positive attitudes, and the practise of positive health advisory behaviours related to COVID-19 prevention procedures. However, high levels of depression, anxiety, and stress were also reported. Overall, women and younger participants were more likely to have high KAP, whereas those living in rural areas and with literacy challenges were less likely to report high knowledge scores.

## Introduction

Spinal cord injury is a common traumatic neurologic disorder that has devastating consequences in social life and is identified as a major health problem in Bangladesh (1). This differently able community has been confronted by multiple challenges and barriers when attempting to re-integrate into Bangladeshi society, which include the following: infrastructure transportation barriers, environmental barriers (slopes and stairs), and physical and socio-economic barriers (2). The key causes of spinal cord injury (SCI) are falling from a height (3); falling when carrying a heavy object on the head (1) and road traffic accidents (3). As the economy of Bangladesh is mainly agriculture-dependent all of these causes can be understood within the cultural context, where young men frequently undertake work with high occupational hazards (1). Due to the unavailability of a central database, the actual mortality rate after having SCI is still unknown but 19% of persons who suffered from SCI died within 2 years of being discharged from the hospital (4). The major complication of severe COVID-19 instances is respiratory involvement. People who have spinal cord injury are more prone to respiratory complications (5) which are similar to symptoms of COVID-19 such as fever (74%), cough (52%), and dyspnea (33%) (6). Furthermore, they frequently have secondary health complications, which increase their susceptibility to SARS-CoV-2 and can be fatal (6). A study published in 2018 states that 6% of SCI patients suffer from severe depression while moderate and mild depression are subsequently 28 and 25.33% (7). As the coronavirus, also known as SARS CoV-2, has caused a larger number of deaths (8), SCI patients who are immunosuppressed (9) are afraid of contracting the virus. Given the identified vulnerability of this community and the recognised major psychological impact of COVID-19 and lockdown periods on the Bangladeshi population (10). Within the general Bangladeshi population, one previous research study examined the KAP relating to COVID-19 within a sample group and found a range of factors were linked to behaviours and the emotional experience of the pandemic (11). Because emotional and immunosuppression statuses are common among SCI patients, determining Knowledge and Attitude is crucial to preventing the spread of COVID (12). Therefore, in this research, an additional aspect was to try to gain some understanding of the overall cumulative psychological impact of the pandemic on the mental health of SCI survivors so that this knowledge could be used to guide and support future care. This research focused on gaining an insight into the knowledge, attitudes, behavioural practises (KAP), and psychological impact relating to COVID-19 in a sample population group receiving in-patient rehabilitation.

## Methods

### Study Design

This study comprised a prospective cross-sectional sample survey of individuals living with SCI in Bangladesh over a 3-month period, who were in active in-patient rehabilitation.

### Setting

The study took place in two tertiary rehabilitation centres in Bangladesh, namely the Spinal Cord Injury Rehabilitation Unit of the Centre for the Rehabilitation of the Paralysed (CRP) and the National Institute of Traumatology and Orthopaedic Rehabilitation (NITOR). In Bangladesh, only these two rehabilitation centres were provided rehabilitation treatment for the people living with SCI.

### Recruitment of Participants

Inclusion criteria for the study were identified as a confirmed medical diagnosis of SCI and required admission to these Rehabilitation centres for active rehabilitation intervention From July to September 2020, all patients admitted for rehabilitation in CRP and NITOR were invited to take part in this study.

This enabled the inclusion of a wide age range of participants. Individuals who agreed and consented to participate in this study were admitted during the said time period to take part in the study. Exclusion criteria of this study were those who were not admitted in the mentioned time period and who did not give their consent, aged <15 years, and second-time rehabilitation or follow-up patients. According to inclusion and exclusion criteria, there were a total number of 207 participants being recruited.

### Sampling Strategy and Estimation

The study used a hospital-based random sampling procedure where participants were selected based on the inclusion and exclusion criteria. From July 2020 to September 2020, a total of 171 patients were admitted at CPR and 117 in NITOR. A total of 153 patients from CRP and 104 patients from NITOR matched the inclusion criteria. However, consent for data collection was given by 149 and 73 patients from CRP and NITOR before starting the data collection, prior permission from CRP and NITOR was taken.

### Study Procedure

**Figure 1:**
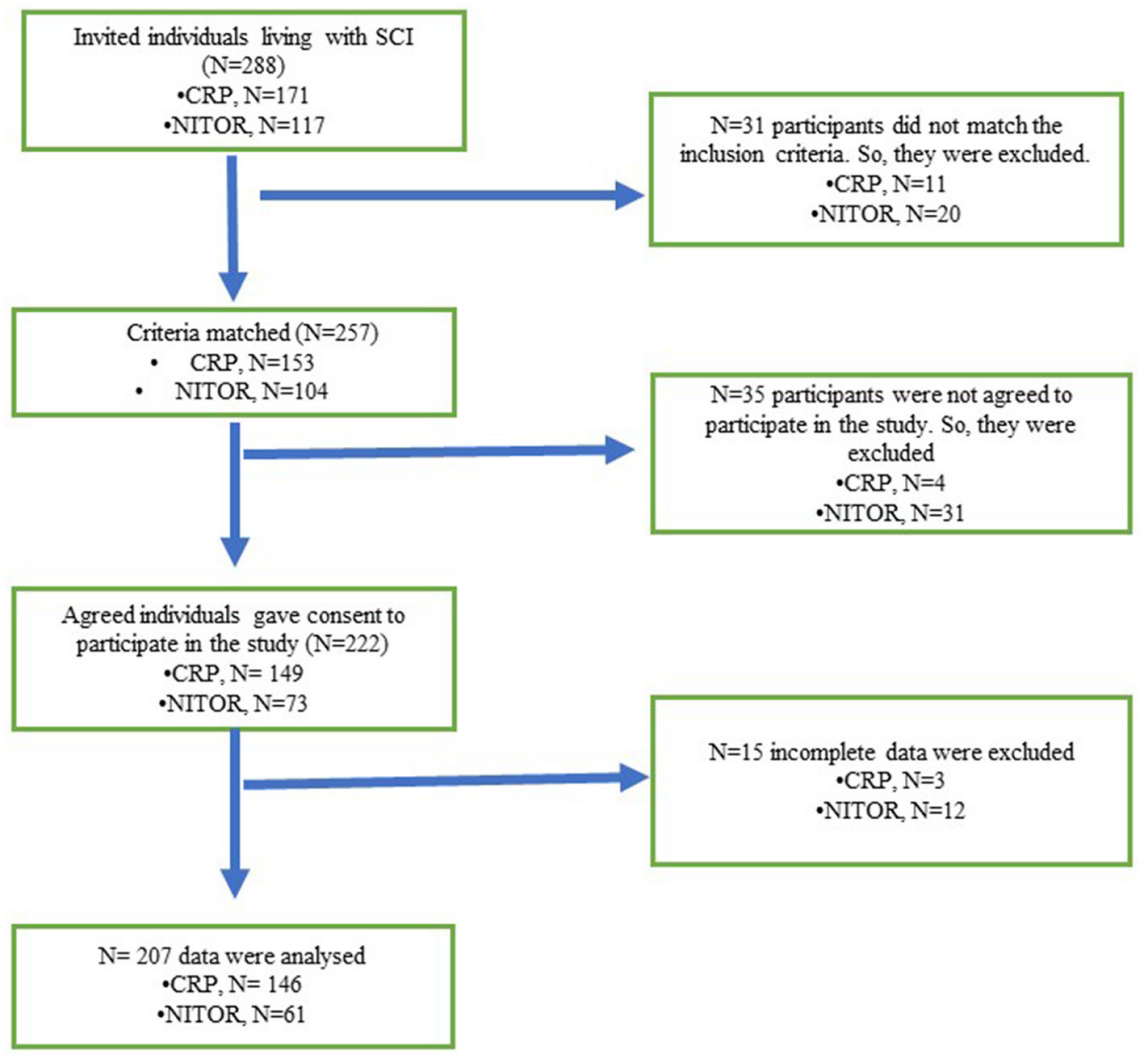
STROBE chart.

A clear flow diagram of the study procedure has been given in Figure 1 to meet the quality by Strengthening the Reporting of Observational Studies in Epidemiology (STROBE) requirements (13). From July to September 2020, a group of trained data collectors collected the data by face-to-face interview and documented the answers given by participants.

### Data Collection Tools

The questionnaire was a mixed type (semi-structured and structured), validated, translated, and customised to fulfil the objectives of the study. American Spinal Injury Association (ASIA) impairment scales A, B, C, D, and E had been used to categorise the patients with SCI (14) which were collected from the record file of hospital patients. The knowledge, attitude, and practise (KAP) questionnaires were 16-score pretested questionnaires taken from a Chinese study (15), customised, translated, and used in a population-based study in Bangladesh (11) where the knowledge section had 12 score questions, attitude, and practise both had 2 scored questions each. There were categorical answers to attitudes and beliefs towards the control of the pandemic, and practises of wearing masks and avoiding public gatherings. The health status and co-morbidities questionnaire (16) (heart disease, blood pressure, respiratory disease, diabetes, ulcer, kidney disease, liver disease, anaemia pressure sore, depression, urinary tract infection, postural hypotension, bowel bladder dysfunction, oedema, muscle spasm, and autonomic dysreflexia) were modified for SCI respondents and taken from a population-based study (17). The depression, anxiety, and stress have been assessed by Depression, Anxiety, Stress Scale (DASS-21), which is validated (18) to use in SCI subjects and had an overall Cronbach alpha score of α0.743 and depression, anxiety, and stress each subscale had α score 0.98, 0.964, and 0.919, respectively, in our study which were indicated satisfactory level of internal consistency with these specific samples. DASS-21 has 7 items for each depression, anxiety, and stress subscales. The sum score of DASS-21 each subscale was divided into cut-off points for the severity rating scale which was also used in a previous study (19). For language validation, Bengali and the linguistic validation process was followed as per WHO guidance (20), first, the questionnaire was translated in forwarding and backward translation to English and Bengali by a group led by a bilingual British researcher. Later it was evaluated and commented on by a bilingual epidemiologist, a psychiatrist, and a clinician experienced in working in SCI-related research.

### Statistical Analysis

The knowledge score was calculated out of 12 scores as per the correct answers provided and considered as continuous data. The attitude and practise answers were categorical data and DASS-21 was calculated separately in depression, anxiety, and stress out of 21 scores; those were continuous data. Descriptive statistics employed in socio-demographic data, to measure relationships among continuous and categorical data-independent sample *t*-test to compare the means for two groups, one-way ANOVA test for categorical data and continuous data. Further Binary logistic regression has been employed using dichotomous variables as the dependent variable and KAP and DASS 21 as covariates. The Pearson test has been used to measure the relationship between two continuous variables. Data analysis was done using the IBM-SPSS version 20. The alpha level of significance was set at *P* < 0.05.

## Results

### Socio-Demographics

The socio-demographic data of the SCI respondents is shown in Table 1. Out of a total of 207 respondents, 180 were men and 27 were women. The mean age of participants was 34.18 ± 12.9 years, with the majority of participants (70.5%) aged between 19 and 40 years and a smaller group (21.25%) were aged between 41 and 60 years. The majority of participants had completed secondary education (27%) and 20% of participants were identified as illiterate. Furthermore, 70% of participants were receiving rehabilitation treatment at CRP and 67% resided in a rural area. Participants resided within and across all the administrative divisions of Bangladesh. The majority of the participants (63%) were diagnosed with an ASIA level of SCI and 137 (66%) individuals were paraplegic, and 70 (34%) participants were tetraplegic.

**Table 1:** Relationship of demographic characteristics with knowledge and DASS-21 scores.

**Characteristics**	**Variables**	**Number of participants**, ***n***	**Knowledge mean ±SD**	**t/F**	**Depression mean ±SD**	**t/F**	**Anxiety mean ±SD**	**t/Chi**	**Stress mean ±SD**	**t/F**
Gender	Male	180	8.46 ± 2.3	2.06^a^ ^*^	11.04 ± 7.8	0.669^a^	7.56 ± 4.9	1.174^a^	9.12 ± 5.8	1.108^a^
	Female	27	9.44 ± 1.6		12.15 ± 9.4		8.81 ± 6.6		10.67 ± 11.2	
Age	18 years and less	12	8.00 ± 2.5	7.905^b^^**^	8.83 ± 6.1	0.523^b^	6.17 ± 3.9	1.596^b^	7.50 ± 4.1	0.603^b^
	19–40 years	146	9.06 ± 2.0		11.25 ± 8.1		7.78 ± 5.3		9.17 ± 7.1	
	41–60 years	44	7.27 ± 2.6		11.32 ± 8.2		8.41 ± 4.9		10.23 ± 6.0	
	>60 years	5	7.80 ± 1.3		13.80 ± 7.8		3.80 ± 2.4		10.20 ± 7.2	
Education	Illiterate	43	8.21 ± 2.0	5.87^b^^**^	11.26 ± 8.3	0.272^b^	7.95 ± 5.2	0.215^b^	8.70 ± 5.7	2.78^b^
	Primary education	54	7.87 ± 2.4		10.28 ± 6.6		7.43 ± 4.9		8.13 ± 4.5	
	Secondary school	56	9.04 ± 2.1		11.86 ± 8.1		7.61 ± 4.6		10.09 ± 5.9	
	Higher secondary	26	8.12 ± 2.3		11.31 ± 7.8		8.46 ± 5.4		7.69 ± 5.4	
	Graduation and above	28	10.11 ± 1.9		11.36 ± 10.1		7.50 ± 6.4		12.57 ± 11.7	
Treatment center	CRP	146	8.53 ± 2.3	−0.528^a^	11.71 ± 8.1	1.45^a^	8.12 ± 5.6	1.69^a^	10.07 ± 7.4	2.48^a^^*^
	NITOR	61	8.72 ± 2.3		9.93 ± 7.2		6.79 ± 3.5		7.54 ± 4.1	
Geographical area of residence	Rural	139	8.58 ± 2.3	5.89^b^^*^	11.60 ± 7.9	3.55^b^^*^	7.60 ± 4.9	1.36^b^	8.86 ± 5.4	7.07^b^^**^
	Semi urban	35	7.69 ± 2.3		8.06 ± 5.3		7.03 ± 3.6		7.60 ± 4.3	
	Urban	33	9.58 ± 1.9		12.76 ± 10.0		9.00 ± 7.2		13.12 ± 11.1	
Administrative Division of Bangladesh	Dhaka	90	8.27 ± 2.1	2.68^b^^**^	9.81 ± 6.6	1.129^b^	7.61 ± 4.4	0.286^b^	8.68 ± 5.3	2.56^b^*
	Chittagong	33	9.67 ± 0.99		11.45 ± 8.3		7.27 ± 5.8		7.09 ± 3.8	
	Rajshahi	7	9.14 ± 4.3		11.71 ± 11.0		7.71 ± 6.0		10.57 ± 5.3	
	Sylhet	9	9.11 ± 1.2		10.44 ± 8.8		7.33 ± 3.4		8.67 ± 6.7	
	Khulna	30	8.59 ± 2.1		13.17 ± 9.7		8.76 ± 6.3		10.48 ± 7.7	
	Barisal	19	8.00 ± 2.8		10.95 ± 5.9		7.68 ± 5.5		9.74 ± 6.2	
	Rangpur	12	9.50 ± 2.3		14.83 ± 11.9		7.00 ± 6.8		15.83 ± 14.9	
	Mymensingh	8	6.75 ± 3.5		13.75 ± 6.7		8.75 ± 3.9		10.50 ± 6.2	
ASIA Diagnosis	ASIA A	132	8.68 ± 2.3	0.212^b^	11.51 ± 8.3	1.457^b^	7.86 ± 5.4	0.239^b^	9.40 ± 7.3	0.458^b^
	ASIA B	10	8.60 ± 2.7		14.80 ± 8.2		8.40 ± 6.7		11.40 ± 7.3	
	ASIA C	45	8.40 ± 2.0		10.49 ± 7.4		7.51 ± 4.4		9.02 ± 5.6	
	ASIA D	22	8.40 ± 2.7		8.80 ± 6.5		7.00 ± 4.0		8.45 ± 4.8	
SCI disability	Tetraplegic	13,770	8.63 ± 2.2	0.110^b^	11.41 ± 8.2	0.360^b^	7.76 ± 5.1	0.018^b^	9.50 ± 7.2	0.286^b^
	Paraplegic		8.51 ± 2.4		10.71 ± 7.6		7.66 ± 5.2		8.97 ± 5.7	

a *Independent t-test*;

b *One-way ANOVA*;

* *Significant with p < 0.05*;

** *Significant with p < 0.01; ASIA, American Spinal Cord Injury Association; CRP, Centre for the Rehabilitation of the Paralysed; NITOR, National Institute of Traumatology and Orthopedic Rehabilitation*.

### Health Status and Comorbidity

**Figure 2:**
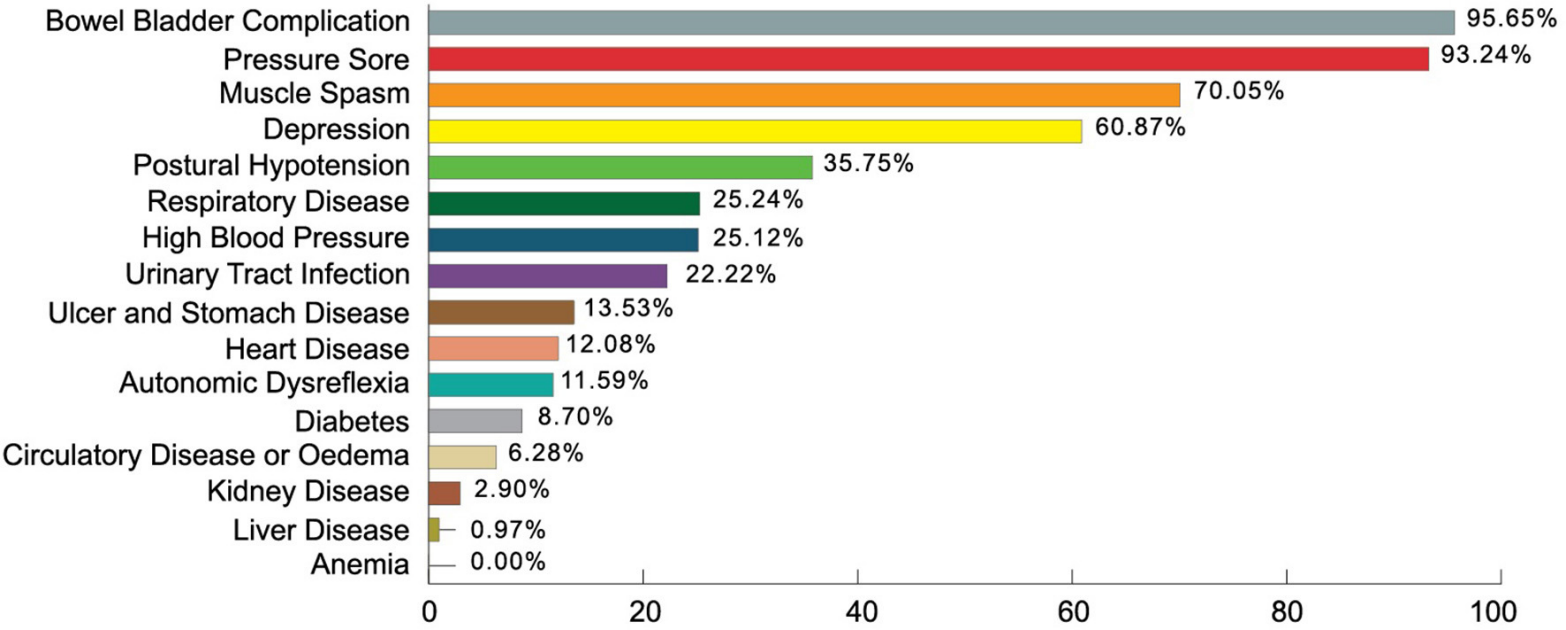
Comorbidities of the participants.

In the International Standard for Neurological Classification of Spinal Cord Injury (ISNCSCI) the mean motor score was 45.38 ± 19.5 out of 100 and the sensory score mean was 97.2 ± 52 out of 253. Oxygen saturation (SpO_2_) mean was 95.07 ± 3.3 and the maximum rate of oxygen consumption (Vo_2_max) found was 35.7 ± 3.7 ml/kg/min. From multiple response analysis, respondents suffering from comorbidities or SCI related complications had bowel bladder complication 95.65%, existing pressure score 93.24%, muscle spasm 70.05%, depressive symptoms 60.87%, postural hypotension 35.75%, respiratory disease 25.24%, high blood pressure 25.12%, and urinary tract infection 22.22% (Details in Figure 2).

### Knowledge

The overall mean knowledge score was 8.59 ± 2.3 out of 12. Women had a greater mean (12.15 ± 9.4) than men (11.04 ± 7.8). There was a higher knowledge score in the age group 19–40 years (9.06 ± 2) but all age groups had a mean of more than 7. The graduates have more knowledge (10.11 ± 1.9) but the illiterate people also have sufficient knowledge of COVID-19 (8.21 ± 2.0). CRP (8.53 ± 2) and NITOR (8.7 2 ± 2.3) have similar knowledge scores, also the knowledge found satisfactory in rural and urban dwellers. Participants from the Chittagong administrative division had the highest knowledge score of 9.67 ± 0.99, then participants from Rajshahi division, Sylhet division, Khulna division, and Dhaka division had knowledge scores of 9.14 ± 4.3, 9.11 ± 1.2, 8.59 ± 2.1, and 8.27 ± 2.1, respectively. On the other hand, the Rangpur division has the lowest knowledge score 2.5 ± 2.3. Participants with the different ASIA diagnostic criteria had nearly similar knowledge scores. Statistical associations were observed with gender and knowledge (*t* = 2.06, *p* < 0.05), age with knowledge (*F* = 7.905, *p* < 0.05), education and knowledge score (*F* = 5.87, *p* < 0.01) and administrative divisions with knowledge score (*F* = 2.68, *p* < 0.01). Details are supplied in Table 1.

In Table 2, binary logistic regression found positive relationships between knowledge scores and gender, wherein men had 6.6 times more knowledge score than women (β = 1.89, odds ratio [OR] = 6.6, *p* < 0.01). Knowledge scores and age group of 19 to 40 years were negatively associated than others, wherein this age group has 0.41 times less score than other age groups (β = −0.879, OR = 0.41, *p* < 0.01). Literate participants and knowledge scores were positively associated wherein literate respondents had 3.8 times higher knowledge scores than illiterate respondents (β = 1.33, OR = 3.8, *p* < 0.01). Respondents from NITOR had negatively associated with knowledge score, wherein knowledge of respondents from NITOR had 0.41 times less knowledge score compared to respondents from CRP (β = −0.873, OR = 0.418, *p* < 0.05), Rural people knowledge scores were negatively associated than urban residency, wherein they were 0.48 times less compared to urban (β = −0.715, OR = 0.48, *p* < 0.01). Tetraplegic respondents' knowledge score was negatively associated, wherein their knowledge score was 0.511 less then paraplegic respondents (β = −0.671, OR = 0.511, *p* < 0.01). Table 3 shows that no statistically significant relationship was found between knowledge scores and family income (*r* = 0.107, *P* = 0.126), depression (*r* =−0.06, *P* = 0.39), anxiety (*r* = 0.04, *P* = 0.49), and stress (*r* = 0.05, *P* = 0.42).

**Table 2:** Results of Binary logistic regression on factors associated with Knowledge and DASS.

**Variables**	**Knowledge**			**Depression**			**Anxiety**			**Stress**		
	**Coefficient**	**OR**	***p***	**Coefficient**	**OR**	***p***	**Coefficient**	**OR**	***p***	**Coefficient**	**OR**	***p***
Male vs. Female	1.89	6.6	0.001^**^	1.89	6.6	0.001^**^	−0.043	0.958	0.243	1.89	6.6	0.001^**^
19–40 years vs. others	−0.873	0.418	0.001^**^	−0.873	0.418	0.003^**^	−0.007	0.993	0.808	0.011	1	0.61
Illiterate vs. literate	1.33	3.81	0.001^**^	1.33	3.8	0.03^*^	−0.011	0.989	0.744	0.019	1	0.49
CRP vs. NITOR	−0.873	0.418	0.013^*^	−0.873	0.418	0.04^*^	−0.055	0.947	0.09	−0.072	0.93	0.01^*^
Rural vs. Urban	−0.715	0.48	0.003^**^	−0.715	0.48	0.04^*^	0.014	1	0.612	0.030	1	0.163
Tetraplegic vs. paraplegic	−0.671	0.511	0.001^**^	−0.671	0.511	0.001^**^	−0.671	0.511	0.001^**^	−0.671	0.511	0.001^**^

* *Significant with p < 0.05*;

** *Significant with p < 0.01; vs., means versus*.

**Table 3:** Relationship among family income, knowledge, and DASS-21 scores.

	**Family income**	**Knowledge**	**Depression**	**Anxiety**
Family income				
Knowledge	*r =* 0.107			
	*P* = 0.126			
	*N* = 207			
Depression	*r=* −0.060	*r =* −0.049		
	*P* = 0.390	*P* = 0.483		
	*N* = 207	*N* = 207		
Anxiety	*r =* 0.047	*r=* −0.041	*r =* 0.452^**^	
	*P* = 0.499	*P* = 0.562	*P* = 0.001	
	*N* = 207	*N* = 207	*N* = 207	
Stress	*r =* 0.054	*r =* 0.058	*r =* 0.586^**^	*r =* 0.476^**^
	*P* = 0.438	*P* = 0.404	*P* = 0.001	*P* = 0.001
	*N* = 207	*N* = 207	*N* = 207	*N* = 207

** *Significant with p < 0.01*.

### Attitudes

The majority of the participants regardless of age, gender and location of geographical residency had agreed on positive attitude (belief in control and world can win) towards COVID-19. Table 4 shows the statistical relationship between education and belief in control (X^2^ = 14, *p* < 0.05); residential living area with belief in control (X^2^ = 9.4, *p* < 0.05) and world can win (X^2^ = 8.84, *p* < 0.05); and geographical area of residence with world can win (X^2^ = 4.19, *p* < 0.05).

**Table 4:** Relationship among attitude and behavioural practices with demographic variables.

**Variables**		**Attitude: Belief in Control**			**Attitude: World can win**			**Practice: Go to crowd**			**Practice: Wear Mask**		
	**Agree**	**Disagree**	**Chi value**	**Yes**	**No**	**Chi**	**Yes**	**No**	**Chi**	**Yes**	**No**	**Chi**
Gender	Male	125	55	3.29	117	63	1.74	58	121	0.38	146	33	0.151
	Female	14	13		14	13		11	17		23	5	
Age	18 years and less	11	1	6.93	10	2	3.47	4	8	4.35	9	3	17.6^*^
	19–40 years	100	46		92	54		50	96		128	18	
	41–60 years	24	20		25	19		14	30		28	16	
	>60 years	4	1		4	1		1	4		4	1	
Education	Illiterate	21	22	14.08^*^	22	21	4.41	11	32	6.0	34	9	11.04
	primary	36	18		35	19		18	36		43	11	
	Secondary school	46	10		40	16		24	32		51	5	
	Higher secondary	15	11		16	10		8	18		17	9	
	Graduation/above	21	7		18	10		8	20		24	4	
Treatment centre	CRP	101	45	0.924	95	51	0.678	52	94	1.45	120	26	0.507
	NITOR	38	23		36	25		17	44		49	12	
Geographical area of residence	Rural	84	55	9.4^*^	79	60	8.84^*^	42	97	6.4	117	22	8.2
	Semi urban	30	5		29	6		14	21		25	10	
	Urban	25	8		23	10		13	20		27	6	
Administrative division of Bangladesh	Dhaka	65	25	1.85	64	26	4.19^*^	38	52	6.4^*^	76	14	2.07
	Other part	74	43		67	50		31	86		93	24	
	ASIA A	83	49	4.33	80	52	3.02	43	89	7.5	110	22	17.9^*^
	ASIA B	7	3		7	3		5	5		4	6	
	ASIA C	32	13		28	17		12	33		36	9	
	ASIA D	17	3		16	4		9	11		19	1	

* *Significant with p < 0.05*.

### Behavioural Practises

The majority of participants (66%) did not go into crowds and wore a face mask during every exposure (80%) (Table 4). 32% of the participants who were men went into a crowd at least once. Furthermore, 18% of participants who were men as compared with 17% of women did not wear a face mask during exposure to outside people. There was a statistically significant relationship found between age and the practise of wearing a face mask (X^2^ = 17.6, *p* < 0.05), geographical location of residence, and practise of solation (X^2^ = 6.4, *p* < 0.05). In addition, there was a statistically significant relationship found between participants diagnosed with complete SCI and the behavioural practise of wearing a face mask (X^2^ = 17.9. *p* < 0.05) (Details are in Table 4). Participants also reported that they and their caregivers followed health advice by consulting health professionals (65.7%), implementing isolation (63.8%), taking droplet precaution care (87.4%), and hygiene care (90.3%).

### Depression

**Figure 3:**
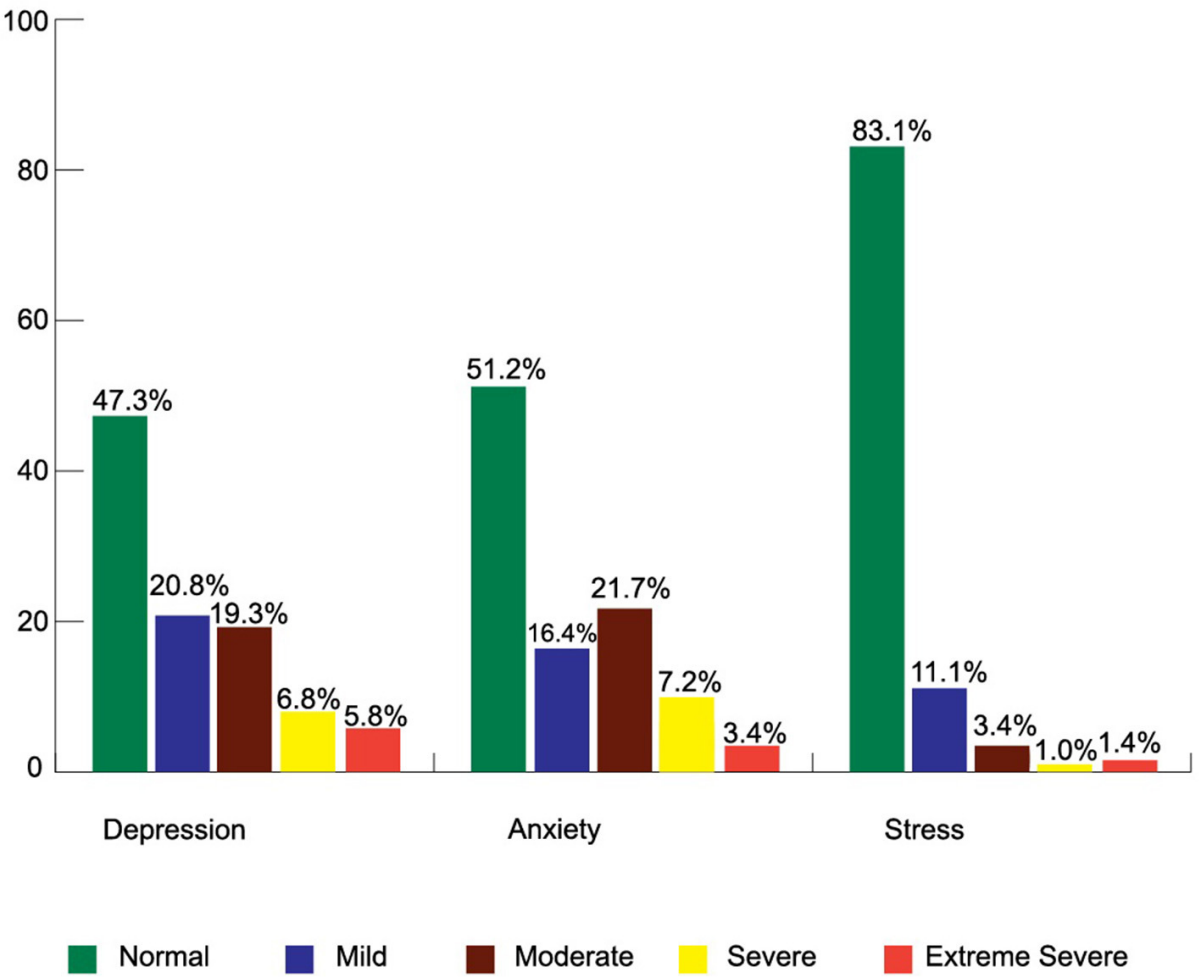
Level of Depression, Anxiety, and Stress (DASS-21) among the participants.

Among the participants, 52.7% had mild to extremely severe levels of depression, whereas 6.8% and 5.8% had severe and extreme depression, respectively (Figure 3). The overall mean depression score was 11.18 ± 8 out of 21. Women had a higher depression score (12.15 ± 9.4) than men (11.04 ± 7.8). Elderly people, more than 60 years old had a higher depression score (13.8 ± 7.8) than other age groups. Urban people had a higher depression score (12.76 ± 10) than rural or semi-urban dwellers. There was a relationship between depression with living area (*F* = 3.55, *p* < 0.05) (Table 1). Table 2 shows that men had a higher depression score compared to women where men respondents depression score was 6.6 times higher than women respondents (β = 1.89, OR = 6.6, *p* < 0.01), and the younger aged people between 19 and 40 years were negatively associated with depression score where 19 to 40 years old participants had 0.41 less depression score than the other age ranges (β = −0.873, OR = 0.418, *p* < 0.01). Literate participants had 3.8 times higher depression scores when compared to illiterate participants (β = 1.33, OR = 3.8, *p* < 0.05) and were positively associated with depression score; Depression score from respondents of CRP was negatively associated, wherein respondents from CRP had 0.41 times less depression compared to the respondents from NITOR (β = −873, OR = 0.418, *p* < 0.05); Participant's residents in rural areas showed negative association with depression scores where rural respondents had 0.48 times less depression compared those residents in urban areas (β = −0.715, OR = 0.48, *p* < 0.05). Tetraplegic participants showed a negative association with depression scores where tetraplegic respondents had 0.51 times less depression than paraplegic respondents (β = −0.671, OR = 0.511, *p* < 0.01). Table 3 represents statistical relationships among depression and anxiety (*r* = 0.45, *p* < 0.01), and depression with stress (*r* = 0.58, *p* < 0.01).

### Anxiety

Among 207 respondents, 48.8% had a different level of anxiety, of which 7.2 and 3.4% reported that they had severe and extreme severe anxiety, respectively (Figure 3). The overall mean anxiety score was 7.72 ± 5.1 out of 21. Table 1 represents women (8.81 ± 6.6) who had higher anxiety scores than men (7.56 ± 4.9). The associated characteristics of participants who had more anxiety were those aged 41 to 60 years, those who had completed higher secondary education (8.46 ± 5.4), those who were resident in urban areas (9 ± 7.8), those who were diagnosed ASIA-B (8.4 ± 6.7), and those with having two comorbidities (8.33 ± 5.3). Binary logistic regression found a negative association with anxiety score and tetraplegic respondents where tetraplegic respondents had 0.51 times less anxiety than paraplegic respondents (β = −0.671, OR = 0.511, *p* < 0.01). Pearson correlation found highly statistical significance (*p* < 0.01) of the relationship between anxiety with depression (*r* = 0.45) and anxiety with stress (*r* = 0.47) (Table 3).

### Stress

Among the participants, 16.9% reported that they had anxiety where 3.4% had a moderate level of stress, 1% had a severe level of stress, and 1.4% had an extremely severe level of stress (Figure 3). The overall mean stress score was 9.32 ± 6.7 out of 21. Women had higher stress (10.67 ± 11.2) than men (9.12 ± 5.8) (Table 1). A statistical association was found between stress with living area (*F* = 7.07, *p* < 0.01) where urban people had more stress scores than others (13.12 ± 11.1). In Binary logistic regression results, men respondents showed a positive association with stress scores whereas men respondents had a 6.6 times higher stress score compared to women respondents (β = 1.89, OR = 6.6, *p* < 0.01) and tetraplegic respondents had shown a negative association with stress score where tetraplegic respondents had.51 times less stress score than paraplegic respondents (β = −0.671, OR = 0.511, *p* < 0.01) (Table 2). In Table 3, a relationship was found between stress and depression (*r* = 0.58, *p* < 0.01) and anxiety (*r* = 0.476, *p* < 0.01).

## Discussion

This survey aimed to gain insight and understanding into the knowledge, attitude, and behavioural practises of people living with SCI towards COVID-19 and determine their anxiety, depression, and stress during in-patient rehabilitation in Bangladesh. The findings indicate that participants had a satisfactory overall knowledge in relation to COVID-19 (8.59 ± 2.3 out of 12), which was similar to the general population in Bangladesh during the COVID-19 period (11). A positive attitude and behavioural practises with regard to listening and the following advice to minimise the risks of catching COVID-19 during rehabilitation intervention. The Centre for the Rehabilitation of the Paralysed (CRP) provided a series of patient education and awareness activities (14) within their rehabilitation interventions during the COVID-19 Pandemic. This indicates that although they are engaged in hospital-based rehabilitation however they received the proper guidelines similar to the general population. Participants reported positive attitudes to the control of COVID-19 and the majority (>80%) followed the health advice provided to them during In-patient rehabilitation. The organisational policies of CRP and NITOR, which aimed to encourage and support the health of In-patients and caregivers might also be a contributory factor towards the positive findings.

The respondents were dominated by men aged 19–40 years, the majority were diagnosed as ASIA-A, were rural dwellers, and with a co-morbidity. This is a common feature as young men living in rural areas are more prone to risks associated with SCI, and 5 years epidemiological study reports that more than 80% of admitted SCIs were men at 55%, with age range 19–40 years, in which 69% lived in rural areas and 60% were diagnosed as ASIA-A (2).

This study revealed the most common co-morbidities such as Bowel bladder complications, pressure soreness, muscle spasms, depression, postural hypotension, and respiratory disease. However, two different studies from Bangladesh found people living with SCI commonly experience co-morbidities including pain, respiratory issues, pressure ulcers, cardiovascular issues, nephrology issues, and physical inactivity issues. Most of the participants were relatively immobile, unable to get out of bed independently, and were reliant on a caregiver for sustaining their survival (3, 4).

Knowledge, attitude, and practise (KAP) questions were validated and used in a population-based survey of 2,157 respondents in Bangladesh (11). Similar findings attributed in that population-based survey, the knowledge score was found higher in women in this study. DASS-21 is validated for SCI cases (18), hence the appropriate process of forwarding and backward translation and expert opinion has been taken before using the tools. The overall depression 11.18 ± 8, anxiety 7.72 ± 5.1, and stress 9.32 ± 6.7 scores from a total of 21 scores seem different from that made with regard to the general population assumption. The study found 5.8% of the respondents had existing issues with extreme depression and the majority of psychological issues were relevant to the gender, young age, and participants diagnosed with complete SCI. Another study from Bangladesh (7) also found almost similar (0.2% higher) SCI people have severe depression during in-patient hospital-based rehabilitation. Hence anxiety and stress are interrelated, and a linear relation was noted between depression and anxiety scores and stress scores. Nearly 61% of Bangladeshi people have stress issues related to COVID-19 which was revealed in a population-based study (21). The anxiety and stress score is likely to be associated with the affordability, safety, and reliability issues of rehabilitation centres not examined in this survey.

There was a correlation between knowledge and DASS with age, as well as between knowledge with gender and education. Binary logistic regression found a higher association of knowledge and DASS with gender, as well as in young age, illiterate, and rural people with knowledge. These findings were identical to the similar population-based study in Bangladesh (11).

There are concerns about the survival, safety, and protection of people living with SCI in the community (18). Future studies of KAP, psychological aspects, adapting and coping with the ongoing waves of COVID-19 infection, and how these impacts on the livelihood of people with SCI living in the community are recommended.

### Strengths and Limitations of the Study

In the perspective of Bangladesh, there is no other study on the knowledge, attitudes, behavioural practises, and psychological impact relating to COVID-19 for vulnerable people with SCI during the COVID pandemic. This study also offers endorsement to the premise that rehabilitation institutions can play an important and effective role in the protection, health promotion, and rehabilitation of vulnerable people, such as those with diagnosed SCI during the COVID-19 pandemic. However, the study population was limited to those who were receiving active in-patient rehabilitation in CRP and NITOR. As a result, the data do not reflect those people with SCI who stayed at home during this time. Because the data was only collected once, there was no way to compare or measure the changes in knowledge, attitude, and practise before and after the rehabilitation phase.

## Conclusion

During in-patient rehabilitation in Bangladesh, the majority of SCI reported that they had communicated with health professionals and practised behaviours that would reduce transmission and risk of COVID-19. The institute-based rehabilitation to SCI was appropriate to protect these vulnerable people from the COVID-19 catastrophe in Bangladesh.

## Data Availability Statement

The datasets presented in this study can be found in online repositories. The names of the repository/repositories and accession number(s) can be found at: https://www.kaggle.com/kmamranhossain/kap-sci-bd.

## Ethics Statement

The studies involving human participants were reviewed and approved by the CRP Ethical Review Committee (CRP-R&E-0401-296). Written informed consent to participate in this study was provided by the participants' legal guardian/next of kin.

## Author Contributions

MH, KH, and RS: conceptualisation, data curation, methodology, formal analysis, and write-up. IJ, MS, and KS: supervision, conceiving, and designing the study and major revisions. RF and LW: data curation and editing. ZU, MK, and MH: input on methodology, analysis, results, and discussion section. MK, MH, and SC: designing the study. All authors contributed to the article and approved the submitted version.

## Conflict of Interest

The authors declare that the research was conducted in the absence of any commercial or financial relationships that could be construed as a potential conflict of interest.

## Publisher's Note

All claims expressed in this article are solely those of the authors and do not necessarily represent those of their affiliated organizations, or those of the publisher, the editors and the reviewers. Any product that may be evaluated in this article, or claim that may be made by its manufacturer, is not guaranteed or endorsed by the publisher.

## Abbreviations

SCI: Spinal Cord Injury
IEDCR: Institute of Epidemiology, Disease Control and Research
CRP: Centre for the Rehabilitation of the Paralysed
NITOR: National Institute of Traumatology and Orthopaedic Rehabilitation
KAP: Knowledge, Attitude and Practise.
